# On Pilot Massive COVID-19 Testing by Antigen Tests in Europe. Case Study: Slovakia

**DOI:** 10.3390/idr13010007

**Published:** 2021-01-07

**Authors:** Jaroslav Frnda, Marek Durica

**Affiliations:** Department of Quantitative Methods and Economic Informatics, Faculty of Operation and Economics of Transport and Communications, University of Zilina, 010 26 Zilina, Slovakia; marek.durica@fpedas.uniza.sk

**Keywords:** nationwide antigen testing, COVID-19, estimation of infection rate

## Abstract

This paper provides a summary of mass COVID-19 testing of almost the entire population in Slovakia by antigen tests. We focused on the results delivered by two testing rounds and analyzed the benefits and weaknesses of such type of testing. We prepared mathematical models to critically examine the effectiveness of the testing, and we also estimated the number of potentially sick people that would become infected by those marked as positives by antigen tests. Our calculations have proven that antigen testing in hotspots can flatten the curve of daily newly reported cases significantly, but in regions with low-risk of COVID-19, the benefit of such testing is questionable. As for the regions with low infection rates, we could only estimate the proportion of true and false-positive cases because the national health authority had not validated the results by RT–PCR tests. Therefore, this work can serve as an introductory study on the first nationwide testing by antigen tests in Europe.

## 1. Introduction

The second wave of coronavirus disease (COVID-19) that had started in October 2020 in many European countries opened the discussion about various strategies for flattening the epidemic curve. Due to the economic impact of the “lockdown” that took place during the spring of 2020, state officials are trying to find more cost-effective solutions. The Central Crisis Staff in Slovakia made a unique decision of a way to beat the virus. This was based on widespread testing of almost the entire population using cheap antigen tests, followed by compulsory self-isolation of positive individuals. This strategy was expected to reduce the spread of COVID-19, especially in hotspot areas, which would result in the decrease of effective reproduction number (mathematically calculated as time-varying reproduction number—$R_{t}$). Mass testing was chosen as the last option to prevent a national lockdown in this country. At the same time, it was the first mass testing of such size in Europe.

A COVID-19 caused by the virus severe acute respiratory syndrome coronavirus 2 (SARS-CoV-2) is not only responsible for a significant number of deaths worldwide, but it also causes economic stagnation or even worse declines in the standard of living. At the beginning of November 2020, the second wave of the coronavirus spread is much stronger than the one we were witnessing in spring. If the pandemic situation worsens exponentially (widespread community transmission), many states will be forced to impose a lockdown (curfew) until the effective reproduction number decreases to a value less than 1. Several studies published during this year pointed to the fiscal impact of moving restrictions on the economy, especially in the manufacturing and services sector (such as tourism or retails) [1,2,3]. Lockdown hits the economy hard and creates serious financial problems for the state budget. The only verified approach that works well as a prevention of the lockdown imposition is mass testing followed by tracing of close contacts of those positively tested. Unfortunately, contact-tracing performed to control the virus spread consumes most of the hygienists’ working time. Testing of the potentially infected individuals (with COVID-like symptoms) and their close contacts are typically made by so-called gold standard diagnostic tests RT–PCR (reverse transcriptase polymerase chain reaction). RT–PCR tests offer high accuracy (almost 100%) [4]. They analyze the genetic information of a given testing sample and search for the RNA of the virus. Virus RNA is present only if someone is actively infected. Although RT–PCR tests are precise, we identify three major disadvantages [5]:

- price (e.g., in Slovakia, one test costs 70 €);
- testing is time-consuming (results are not available within 24 h);
- patients who recovered from COVID-19 are identified as positives by RT–PCR for a long time after the symptoms have waned.

Ongoing development in testing has brought two alternatives. The first one is an antibody test, which checks if the tested person has antibodies in their blood. Tests provide very fast results and answer the question if the tested person has had a virus. However, these tests are not commonly accepted by authorities because they do not recognize if the person is infectious (infectiousness has dissipated, but the test is positive). Another, more popular type of rapid diagnostic test is called the antigen test. Antigen tests detect virus activity in the form of viral proteins (antigens). These tests are suitable for identifying individuals that have the virus in the infectious stage when it reproduces quickly. The key benefit of these tests lies in the ability to prevent further infection caused by gathering with a larger number of people. The test can deliver results very quickly (15–30 min), and the price oscillates between 4 and 5 € per test [6,7]. The reason why these tests have not been widely used for mass testing is their sensitivity parameter. According to the clinical studies [7,8,9,10], in an uncontrolled environment, the sensitivity can drop sharply to less than 80%, which would result in a huge number of positive persons that receive false-negative results. However, for the countries which test only symptomatic people by RT–PCR, sensitivity has not played such a crucial role. Asymptomatic people (some published results declare that almost 80% of all infected individuals have no or mild symptoms [11,12]) are not included in the official statistics because rules of the national testing policy do not apply to them. However, in fact, they participate in infection propagation, too. Another parameter, specificity, describes how many people without COVID-19 infection would be marked as negative. If testing sends an unnecessary number of participants to home isolation, a decrease in their income may make them become less willing to abide by the state’s restriction policy (travel restriction, social distancing, closed shops or restaurants), or it will lead to the necessity to increase state compensation for these people. Antigen tests achieve better results in their specificity parameter (97.6–99.3%) [13].

In this section, we must point out the important limitation of antigen tests. Even though Slovakia (a Central European country), with a population of five and a half million, has seen the number of infected people grow rapidly as a whole during October, the situation is varying in the individual regions. In a population where we expect a very low prevalence (less than 1% [9,14]), false-positive individuals can occur more frequently than really infected ones, even when using a test that declares 99% specificity. False-negative cases can be discovered in the population by repeating the test (e.g., by retesting a week later). False-positive cases can be confirmed only by RT–PCR retesting, which cannot be carried out in the situation of mass testing. However, it is correct to say that countries facing high COVID-19 transmission in a certain local community should consider testing the whole population within that affected area. This would enable the identification of infectious COVID-19 cases, and it would allow immediate self-isolation to stop the virus from spreading.

Despite all the above-mentioned facts, the Slovak government has ordered antigen tests from three companies (Biosensor Standard, RapiGEN, and Abbott) [6] and divided the testing strategy into four phases:

- trial testing (in four critical districts);
- nationwide testing (includes retesting of four districts from the trial round);
- testing of incoming travelers at the state borders;
- mobile collection points in every district—offering free antigen testing.

Till the time of writing this text (November 2020), mass testing of the population has only been performed in China, where the whole city of Qingdao (nine million people) was tested within three days [15]. In addition, in May 2020, the world epidemic center of COVID-19—the city of Wuhan—was continuously retested. In Europe, we have not had any record of nationwide mass testing within the state or at least testing of more than a million people per day by antigen tests.

This paper is trying to bring a comprehensive overview of this unique mass testing of almost the entire Slovak population. We have analyzed the results delivered by the first and second rounds of testing and prepared an analysis of its effectiveness in the form of mathematical simulations. Our models estimate the total number of people that hypothetically get infected by those who were discovered by antigen testing. We have also investigated how potentially infected people would reduce the quantitative requirements for hospital treatment, including the number of inpatients and intensive care unit (ICU) beds. Last, but not least, we calculated the probability of being in close contact with an infected person during the testing procedure, because as everybody knows, during these difficult times—safety first.

## 2. Methodology

As a starting point, the four most affected districts in Slovakia were chosen for trial testing (23–25 October 2020). This round primarily served as an initial stage for nationwide testing that was about to be performed during the next weekend (two days). Every citizen aged between 10 and 65 had to visit one of 182 testing points or stay in home quarantine for 10 days (curfew). Slovak government had bought antigen tests that had to meet minimal criteria defined as follows: specificity higher than 99% and sensitivity higher than 93% (in laboratory conditions) [6]. Slovakia had already announced a state of emergency at the beginning of November and implemented restrictions to reduce the community virus spread, such as the mandatory wearing of face masks outside, food and beverage service providers can operate only via delivery, serving hatches or outdoor seating service. All public events are now allowed.

Collection points for sampling during the COVID-19 testing were situated mostly in schools and local government buildings, with an emphasis on having outdoor waiting areas (e.g., school playground). All participants had to wait for their results delivered in the form of a medical certificate. The testing process was secured by military forces, medical staff and public servants. Each collection point was dimensioned to carry out 330–350 tests per day. The interval between the two tests was approximately 2 min (30 tests per hour), and a maximum of 15 people could be in the waiting area at the same time (with social distance at least 2 m). Unfortunately, we have no data on the combination of tests from the selected distributors that were used for each testing. Since we wanted to create prediction models for estimation of the number of potentially positive cases (that would be infected by the ones discovered during the trial round), we designed a realistic and so-called less accuracy-testing (LAT) scenario (based on documented specificity of the tests). We carefully read the above-mentioned studies, which have proven that accuracy receives considerably lower value outside than in the laboratory conditions. In a realistic scenario, we set specificity to 99%, while the LAT scenario counted with specificity 97.5%. Sensitivity was not an important parameter for modeling because we had no possibility to identify false-negative cases.

### 2.1. Mathematical Models for Estimation of Infected People within the Population

There are two commonly used models on how to describe virus behavior within a population. The first one is the exponential model. The exponential model estimates an uncontrolled disease expansion. The basic formula can be defined as follows [16,17]:

(1) $$f\left(x\right)=ae^{b\left(x-c\right)}$$

where a stands for the initial value of the model, x represents the day of the prediction and b(x−c) represents the growth factor. Another mathematical model we used was the logistic model, defined by the second equation [15,16]:

(2) $$f\left(x\right)=\frac{c}{1+e^{-\left(x-b\right)/a}}$$

In this formula, a refers to the infection speed, b could be considered as incubation rate, c denotes an upper boundary of cumulative positive cases estimation and finally, x stands for the day of prediction.

### 2.2. Was the Mass Testing Safe for People?

We also focused on risk analysis—the probability of being close to (meet) a potentially infected person during the testing procedure. Trial testing showed that people were exposed to a risk of infection due to spending approximately 1 h at the testing place (waiting in the queue, taking samples, waiting for results). By using exponential distribution, the probability that at least one individual is infected in a group of a size n (number of citizens that are in the common area at the same time) is calculated in the following way:

(3) $$\frac{t_{1}}{t_{t}}\int_{0}^{1}\int_{0}^{y+1}\lambda e^{-\lambda x}dx\;dy+\frac{t_{2}}{t_{t}}\int_{0}^{2}\lambda e^{-\lambda x}dx$$

where $t_{1}$, $t_{2}$ represent a specific part of collection point operating hours (see explanation below formula 4), $t_{t}$ is a total testing time in hours per day, and λ is given as:

(4) $$\lambda =\frac{N_{ep}}{N_{d}t_{t}}$$

where $N_{d}$ stands for the total number of testing days and $N_{ep}$ represents the total number of expected positive cases that visit one testing point. The integral boundaries were set in accordance with the following situations: in case of attending the collection point immediately after its opening, a participant can meet only with those who arrive within the next 1 h after him. A similar situation happens at the end of the testing day—a participant can meet only those who have already arrived a maximum 1 h before him (both cases are represented by interval 0–1). We also incorporated the 1 h break between the morning and afternoon opening hours of the collection point, which also results in the same situation as described above thus $t_{1}$ represents the number of hours (in our case four) of limited contact with others during the testing procedure. Double integral reflects on the situation when the participant visits the collection point within the first hour from the start of the testing (or if there is less than 1 h left to the end of the testing period). In this case, parameter y lies in the open interval (0,1).

If a participant gets tested any other time, they can meet with those who came before him (maximum 1 h), as well as with those who came after him (the testing procedure takes one h). This situation is represented by the interval from 0 to 2 and took $t_{2}$-hours of one testing day (in our case 7 h).

## 3. Results

**Figure 1:**
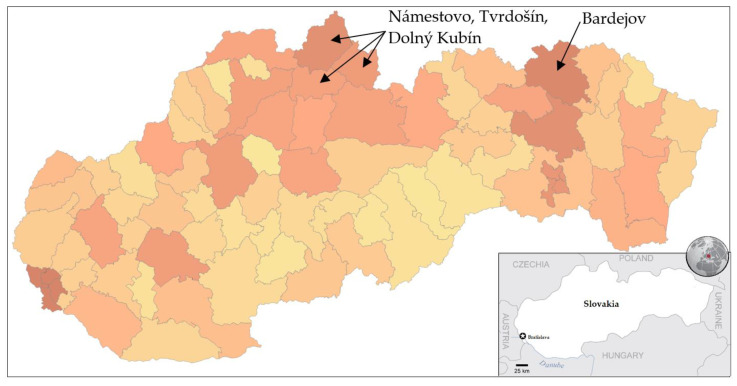
Pandemic situation on the day before the trial testing. Dark color characterizes districts with a higher number of confirmed positive cases by RT–PCR tests [18].

Trial testing as a part of nationwide testing was performed in four most affected districts of Slovakia, namely Tvrdošín, Námestovo, Dolný Kubín and Bardejov. The geographic location of these districts is depicted in Figure 1. Testing turnout was 90.93%.

Testing was mandatory for all citizens older than 10 years, whereas citizens above 65 who did not go to work did not must participate in testing. Generally, all those nonparticipants had to stay in self-isolation for the next 10 days. Epidemic situation on the day before testing is stated in Table 1. The nation’s seven-day average of daily new COVID-19 cases growth rate oscillated from 25% to 35% [18].

A total number of 140,945 participants were tested during the trial round. Test positive percentage reached 3.97%. In comparison to the traditional RT–PCR testing also performed in these districts, eight times more people were identified as positive (5 594 vs. 782) by antigen tests. All interesting results obtained from the trial round are shown in Table 2.

We have no data on the number of RT–PCR tests performed in these districts; the Ministry of Health only provides the number of positive cases for each district in Slovakia. Trial testing showed that the prevalence in these districts was relatively high, and many positive cases discovered by antigen tests would probably not be caught by RT–PCR tests. Since the sensitivity of antigen tests is a hot topic in many research papers, we assume that the incidence in the population will be higher than trial testing has shown, more specifically somewhere between 4 and 5%. Based on this assumption, we operated with a prevalence of 5%. In a realistic scenario (99% specificity), we estimate 4255 true positive cases (76%; first day: 1692, second day: 1679 and third day: 884) with 1339 false-positives out of 5594. In our LAT scenario (97.5% specificity), the number of identified test positive cases is 2247 (40%; first day: 894, second day: 887 and third day: 467), thus 3347 false-positive cases out of 5594 per the given tested population. In this population with a prevalence of 5%, the number of positive tests would be 7047 (6265 without RT–PCR positives), so it means that antigen testing identifies (estimation) from 36% (LAT) to 68% (realistic scenario) of all infected individuals in the population. According to these scenarios, the RT–PCR testing policy in Slovakia was able to trace approximately 11% of all infected individuals in the population for the given period. A similar situation was in China, Italy or in the USA, where scientists projected that the number of confirmed cases in hotspots could oscillate between 10 and 20% of all infected people [21,22,23].

In addition, an interesting question emerges: How safe is the testing? Can I meet someone infected by COVID-19 during the testing procedure? Let us make the following assumption: For example, one collection point can process approximately 1000 individuals per the whole testing period. The prevalence is 5%, the testing procedure takes 1 h. Thirty individuals are tested per hour, and the total testing period is three days (11 h per day). What is the probability that I will be in close contact with someone who is infected? If we put all this information into Equations (3) and (4), we will get an answer. If I am healthy, the probability that during this one h I meet someone infected is 92.8%. It has been verified that wearing a face mask and keeping a social distance both are important means of self-protection against the virus spread.

**Figure 2:**
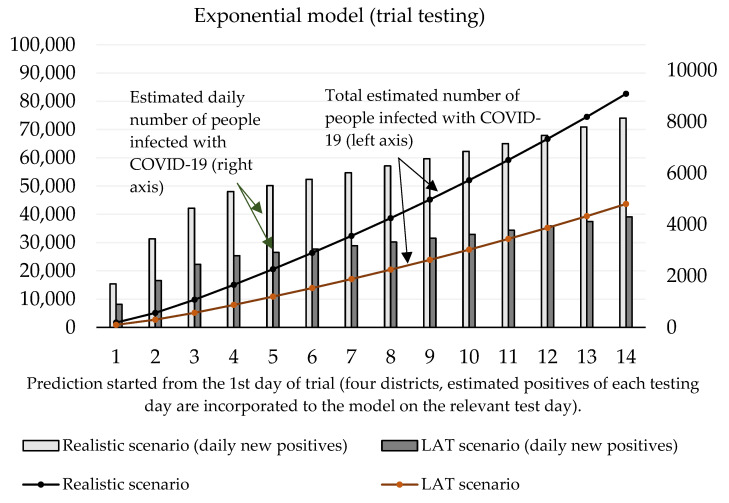
Forecast of the cumulative number of infected people (caused by those who would not be discovered by trial testing) based on the exponential model.

**Figure 3:**
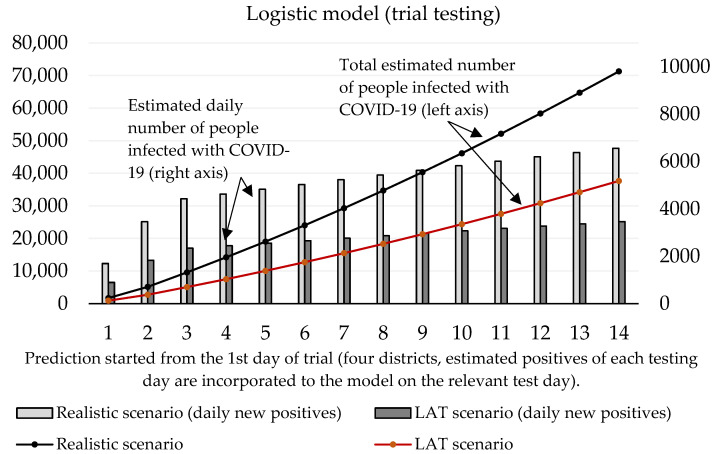
Forecast of the cumulative number of infected people (caused by those who would not be discovered by trial testing) based on the logistic model.

Now we can predict the daily number of newly infected people caused by those who were revealed by trial testing. The number of positive cases at the initializing phase is given as the estimated true positive cases from the first testing day (according to the ratio between the total number of positive test cases and our estimated number of true positive cases). On the second and third day of the trial round, our forecast takes into account the model’s predicted numbers, as well as the amount of discovered daily true test positive cases for these two days. The values of coefficients introduced in Section 2.1 are shown in Table 3 and Table 4. Prediction of the cumulative number of infected cases for the following 14 days is depicted in Figure 2 and Figure 3.

For the logistic model, estimating the growth rate plays a crucial role. It is important to find a credible function because the number of daily infected people will decay with time. We incorporated information about the growth ratio of the nation’s 7-day average of daily new COVID-19 cases over the past two weeks. For logistic prediction, we used the decay model that the number of daily new positive cases will increase by 30% (comparison between 1st and 7th day) and by 25% (comparison between 7th and 14th day of the forecast). The exponential model was set to a 35% increase (comparison between 1st and 7th, and 7th and 14th day of the forecast). These settings reflected on the situation before the trial test was performed. The estimated daily number of true positive cases identified by antigen testing was incorporated into a given test day of prediction.

**Figure 4:**
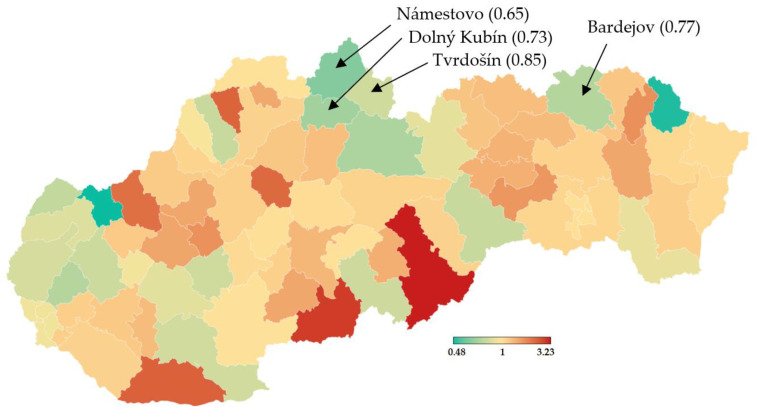
Multiplication of 7-day incidence- RT–PCR testing (28 November vs. 21 November) [20].

According to the official government statistics depicted in Figure 4, a week after the trial, the 7-day incidence dramatically dropped in the districts chosen for the initial testing round [20].

Figure 3 shows that according to the predictions of the logistic model, the cumulative number of people hypothetically protected from infection due to them not meeting individuals classified as positive in trial testing on day 10 ranges between 24,369 (LAT) and 46,137 (realistic). If we set the hypothesis that RT–PCR tests confirmed 11% of them and approximately 2.6% (as of 7 November) [18] of the reported cases would need to go to the hospital (70–132), then 5 to 10 (7.6% of hospitalizations) of them would need intensive care units (ICU). The exponential model delivered slightly worse numbers: the cumulative number of hypothetically infected people ranges between 27,510 and 52,060 (10th day of the forecast). Hospitalization would be necessary for 79–149 patients, and the availability of ICU beds would be reduced by 6–11 beds in Slovak hospitals.

**Figure 5:**
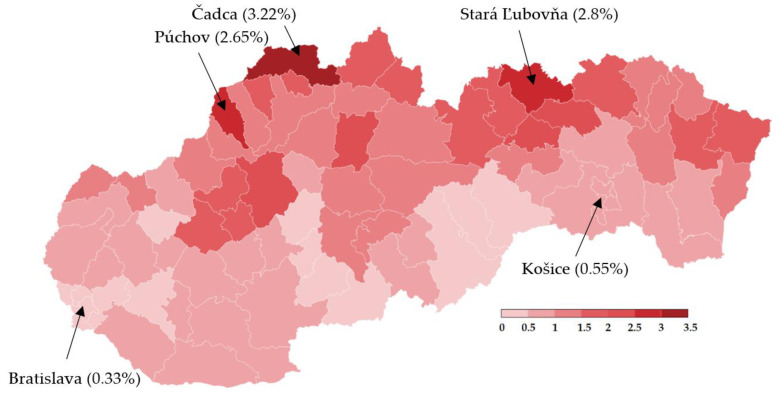
Test positivity percentage in Slovakian districts (mass antigen testing 31 October–1 November 2020) [20].

Nationwide testing was performed one week later for 2 days. More than 3.6 million Slovaks visited the collection points. Test positivity percentage reached a value slightly over 1%, as shown in Table 5. Separately, RT–PCR tests discovered another 4165 positive cases. The positive test rate was varying widely across the districts in Slovakia. This testing identified another three hotspots with more than 2.5% of positive cases (Čadca, Púchov, and Stará Ľubovňa). On the other hand, there were many districts with positivity below 1% (e.g., the capital city Bratislava and the second biggest city Košice). For each district, the test positivity percentage is depicted in Figure 5. Nationwide testing detected a very low prevalence in almost half of the country. According to the WHO [19], antigen tests should not be used for population screening, especially if the expected prevalence is less than 1%. There is an assumption that a high portion of positive cases can be false-positives. Unfortunately, without the RT–PCR verification of all the positive test cases, it is not trivial to forecast the ratio between true positives and false-positives.

**Figure 6:**
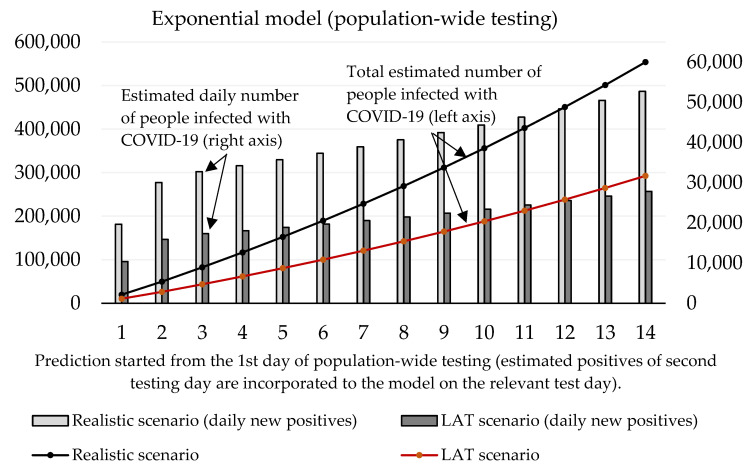
Forecast of the cumulative number of infected people (caused by those who would not be discovered by nationwide testing) based on the exponential model.

**Figure 7:**
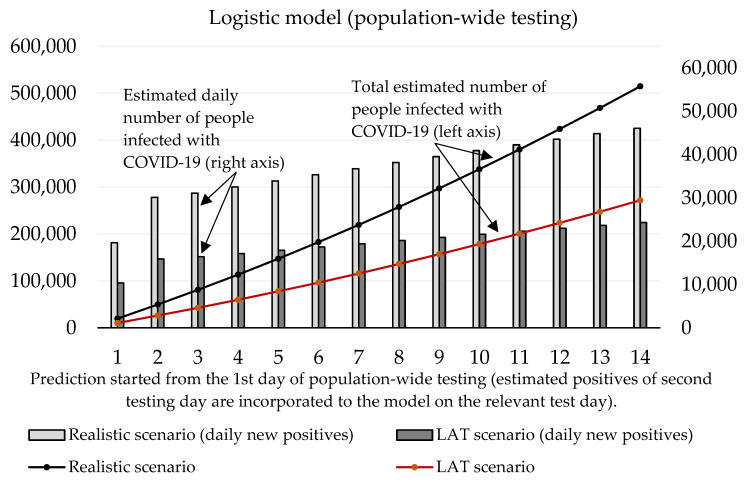
Forecast of the cumulative number of infected people (caused by those who would not be discovered by nationwide testing) based on the logistic model.

With regard to these results, as well as to the prediction made by the Ministry of Health of Slovakia, we can say that the expected prevalence in the population is 1.5%. Under the circumstances, a real total number of active cases in the Slovak population should achieve a value of 55,000. After the exclusion of RT–PCR positive tests, the total number of infected people able to be detected by antigen tests is 51,000. Our estimation of daily new positive cases is depicted by Figure 6 exponential model and Figure 7 logistic model. By using the same conditions as during the trial testing, we expect that antigen tests recognized from 40% (LAT) to 76% (realistic scenario) of true positive cases from the test positives. In absolute numbers, models estimate from 15,409 (1st day: 10,384, 2nd day: 5025) to 29,176 (1st day: 19,662, 2nd day: 9514) positive cases. Hence, we postulated that laboratory tests covered 12% of all COVID-19 infected people (almost the same value that was calculated for the trial testing).

Both models predict that mass testing helped to reduce the surge of new cases. Compulsory home quarantine of test positives aims to reduce the number of people they could infect. Our mathematical models estimate the total cumulative number of sick people hypothetically infected by antigen test positives as follows (10th day of the forecast):

- Logistic model: 178,612 (LAT) to 338,186 (realistic);
- Exponential model: 188,087 (LAT) to 356,140 (realistic).

In a detailed view, these numbers as active reported cases (12% of the infected population would be recognized as active cases caught by RT–PCR in official reports) would send 557–1055 (logistic model) or 587–1111 (exponential model) sick people to a hospital within the next 2–3 weeks since the start of the prediction. Some of them would need ICU, and based on our models; we estimate that 42–80 (logistic model) or 45–85 ICU beds should be occupied additionally by these COVID-19 patients (as of 7 November, 121 patients are admitted in an ICU in Slovak hospitals [18]; thus we predict that without the mass testing the ICU bed occupancy could be almost doubled in the worst-case scenario).

## 4. Discussion

Mass testing of almost the entire population of Slovakia was a unique operation in Europe. Results obtained from two rounds of testing (trial testing in four districts and nationwide testing) have fully revealed all benefits and weaknesses of this type of testing. These tests are cheap and provide rapid diagnostic results in comparison to laboratory performed RT–PCR tests. Although both sensitivity and specificity are lower, antigen tests help to recognize many infections spreading individuals with no or mild symptoms and protect others from becoming sick of COVID-19. As the first step, the state government identified the hotspots and chose four districts for the trial round, as depicted in Figure 1. Hotspot regions were recognized by their 7-day average incidence of coronavirus (COVID-19) cases over the past four weeks provided by the results of RT–PCR tests. All the selected districts were located near the state borders with Poland and the Czech Republic, where the pandemic situation was (at that time) worse than in Slovakia, but borders were still open. All people above age 10 had the option to participate in testing or self-isolate themselves for ten days at home (as well as test positives). Any quarantine-breaker could face a fine (1650 €). Because the expected prevalence was high in the hotspots, we also calculated the probability of meeting someone infected during the testing procedure, which took approximately 1 h (waiting in a queue, testing, and waiting for the result). In these regions, the probability of meeting the infected individual was approximately 92.8%.

Table 5, as well as Figure 4, prove that one week after the trial round, the prevalence in the hotspots dropped dramatically. It resulted in reducing hospital bed occupancy (models predicted up to 150 hypothetically new hospitalizations for the first 10 days of prediction). Therefore, we can say that antigen testing in these four districts had its meaning, especially in the light of statistics on the national level. Seven days after the trial, $R_{t}$ increased from 1.2 to 1.25, and the 7-days rolling median of positive cases increased from 1728 to 2887 (529 per 1 million) [18]. Thus, except for the four tested districts, the epidemical situation in Slovakia got worse (comparison of weeks 43 and 44).

The nationwide screening was performed during the weekend days on calendar week 44. One week later (Week 45), the results were optimistic. Although the incubation period for COVID-19 ranges from 4 to 7 days on average, when compared to Week 44, $R_{t}$ decreased to 1.1, and the 7-day rolling median of positive cases dropped significantly to 2282 (418 per 1 million). Nationwide testing of almost the entire Slovak population was a massive operation that required the necessity of cooperation between the military forces (security and construction of the collection points), medical staff and local municipalities (provided locations for collection points and financial support for buying of protective equipment for the staff).

A report from the Ministry of Health related to Week 46 says that the effective reproduction number was below 1 for the first time since the beginning of the second wave ($R_{t}=0.7$) and the 7-days rolling median of positive cases dropped again to the value 2024 (371 per 1 million) [18]. This report was presented at the press conference (13 November), during which the minister said an interesting fact: without the nationwide testing, they estimated at least 4000 daily new RT–PCR test positive cases per day at the beginning of November [24]. A day later, the Slovak Prime Minister informed that national health authorities predicted 5000 new daily reported cases for the second week of November if the nationwide testing had not been realized [25]. If we have a look at our prediction, we can provide a simple validation. For example, our logistic model uses a constant 4.85 as an incubation period. Therefore, the number of estimated RT–PCR test positive cases were related to the number of infected individuals five days ago. The realistic scenario predicts 3738 (number of new cases for the third day of the forecast–31,115 × 0.12) daily new positive cases confirmed by RT–PCR on the 8th day of the forecast (7 November). For a week later—14th day (13 November), the model estimates 4746 (number of new cases for the ninth day of the forecast–39.554 × 0.12) positive cases reported by RT–PCR tests. We also must take into account the uncertain portion of hidden cases (false-negatives coming from antigen testing) that could be confirmed by RT–PCR testing. However, we can say that we have prepared a short-term prediction that should reflect the real situation with the virus spreading, and model outputs are reliable.

On the other hand, in very low affected regions, the credibility of delivered results is questionable because of (hard to estimate) a high portion of false-positive cases. We have identified the absence of verification of antigen-positive cases by RT–PCR tests as the major weakness of this mass testing. We can only estimate the true proportion of positives based on antigen test specifications. Likewise, we did not measure the prediction error of our forecast because the situation after the mass testing has changed significantly; thus, we did not have target (expected) data for the model output validation. We also did not calculate standard error or confidence intervals because our model belongs to time-series prediction, where each day contains only one unique value of positives cases. On the other side, more than 3.6 million Slovak citizens participated in this testing. Health authorities got the approximate number of prevalence at the national level, which allowed them to make additional decisions about the repurposing of the hospital beds.

At the same time, we must consider the false illusion of being negative (antigen tests have lower sensitivity than RT–PCR). We fully understand that RT–PCR tests cannot be used in such volumes (we estimate that laboratory tests have discovered only 11–12% of all infected people in the population), but we recommend verifying antigen test positive cases by laboratory testing, especially in very low prevalence regions, or do not use mass antigen screening of population in areas with expected prevalence under 1% at all. We suppose that Slovak Prime Minister Matovič made a political decision on his own and took responsibility for the testing of almost all Slovak citizens. However, the results obtained from the mass testing have proven that in regions with a very low expected prevalence, mass testing is ineffective and unworthy of its financial and human capital costs (approximately 20,000 medical staff and 40,000 non-medical staff participated in two rounds of testing). Ministry of Defense assessed its costs for about 30 million euros, plus additional 52 million euros were spent on purchasing 16 million antigen tests to run the testing program [20].

We consider it a good solution to test (almost) all individuals that want to cross the borders because moving restriction and social distancing have less impact on flattering the curve of daily cases if travelers with unknown travel history can move as they want with a high-risk of importing COVID-19 disease to the country.

## 5. Conclusions

This paper provides a summary of the first nationwide mass testing by antigen tests in Europe. We proposed mathematical models trying to estimate the impact of self-isolation for test-positive cases which would (probably) not be tested by RT–PCR due to their asymptomatic sickness. Our prediction has proven that in hotspots, antigen testing can significantly slow down the spread of illnesses. In regions with low positivity expectation, positive cases marked by antigen tests should be retested by RT–PCR for validation. However, in general, nationwide testing by antigen tests has identified many potentially infected individuals within the population that would not be discovered, yet they would be involved in the spread of the virus, causing COVID-19. All public data related to the past and ongoing epidemical situation in Slovakia is available here [26].

## Figures and Tables

**Table 1:** Official measures are taken by the Ministry of Health of the Slovak Republic related to the day before the trial testing (22 October 2020) [18].

7-Days Rolling Median of Positive Cases	Active Cases	Effective Reproduction Number $R_{t}$	Total Number of Recovered Patients	Total Number of Deaths Caused by COVID-19	Mortality Ratio in%
1728 (316 per 1 million)	28,918	1.2	8859	134	1.49 ^1^

^1^ Calculating as case fatality ratio during an ongoing epidemic, according to the World Health Organization (WHO) [19].

**Table 2:** Summary of trial testing (23–25 October 2020) [20].

Number of Collection Points	Eligible for Antigen Testing	Number of Tested People	Total Number of Positives	Test Positive Percentage
812	155,000	140,945	Antig. tests: 5594	3.97%
			RT–PCR: 782 ^1^	NA

^1^ Total number of RT–PCR test positive cases (tested separately) during the testing period in four districts [18]. Theoretically, there is a possibility that someone who has been tested by PCR tests also visited one of the collection points for antigen testing on the same day or during the following days. However, we have no information on this.

**Table 3:** The coefficients table for the exponential model.

Round of Testing	Model	a	b	c
Trial testing	Exponential	1692/4642 ^1^	0.04325	0.999
	Exponential (LAT)	894/2453 ^1^		
Nationwide testing	Exponential	19,662/30,046 ^1^	0.04325	0.999
	Exponential (LAT)	10,384/15,868 ^1^		

^1^ Initial model value/for daily forecast after the testing period.

**Table 4:** The coefficients table for the logistic model.

Round of Testing	Model	a	b	c
Trial testing	Logistic	11.9	4.85	4030/9590 ^1^
	Logistic (LAT)			2130/5060 ^1^
Nationwide testing	Logistic	11.9	4.85	46,835/67,463 ^1^
	Logistic (LAT)			24,735/35,630 ^1^

^1^ Initial model value/for daily forecast after the testing period.

**Table 5:** Summary of nationwide testing (from 31 October–1 November 2020) [20].

Number of Collection Points	Eligible for an Antigen Testing	Number of Tested People	Total Number of Positives	Test Positive Percentage
4912	3,800,000	3,625,332	Antig. tests: 38,359	1.06%
			RT–PCR: 4165	9.59% (43,426 ^1^ RT–PCR tests)
**Excerpted results dedicated to the districts of pilot testing**				
812	155,000	123,896	Antig. tests: 2122	1.71%
			RT–PCR: 107	NA

^1^ Total number of RT–PCR laboratory tests performed separately during the testing period [18].

## Data Availability

The data presented in this study are openly available in GitHub Repository, reference number [26].
